# Impacts of Farrowing Pen Design, Season, and Sow Parity on Litter Performance and Piglet Mortality

**DOI:** 10.3390/ani14020325

**Published:** 2024-01-20

**Authors:** Verônica Madeira Pacheco, Tami M. Brown-Brandl, Gary A. Rohrer, Rafael Vieira de Sousa, Luciane Silva Martello

**Affiliations:** 1 Department of Biosystems Engineering, University of Nebraska-Lincoln, 3605 Fair St., Lincoln, NE 68588, USA; veronica.pacheco@unl.edu; 2 United States Department of Agriculture (USDA)—Agricultural Research Service, US Meat Animal Research Center, Clay Center, NE 68933, USA; gary.rohrer@usda.gov; 3 Department of Biosystems Engineering, Faculty of Animal Science and Food Engineering (FZEA), University of São Paulo (USP), Av. Duque de Caxias Norte, 225, Pirassununga 13635-900, SP, Brazil; rafael.sousa@usp.br (R.V.d.S.); martello@usp.br (L.S.M.)

**Keywords:** alternative farrowing pen layouts, piglet mortality, piglet performance traits, parity, season

## Abstract

**Simple Summary:**

Pre-weaning mortality represents a significant concern for pig producers, impacting both animal welfare and productivity. Factors impacting piglet performance include housing systems, parity, and season. While several studies have investigated different sizes of traditional farrowing pens, comparisons between diverse layouts or space utilization are lacking. This study aimed to assess the impact of different farrowing pen layouts on piglet performance, considering the influences of season and parity. The results suggest that modifying the layout, with sows placed farther from the heating source, can reduce the percent of overlays in high-mortality sows (>2 piglets). Additionally, the study revealed that the litters from first-parity sows had a lower average daily weight gain compared to those from multiparous sows. Seasonal variation also influenced production parameters (percent of overlays and average daily weight gain). These findings demonstrate the importance of proper environmental management, even in systems with a certain level of climatic control.

**Abstract:**

Piglet mortality during lactation is a significant concern in swine production, influenced by complex interactions involving sow, piglet, environmental, and management factors. While crushing by the sow may be the ultimate cause of piglet mortality, there are many factors influencing the outcome, including parity, thermal stress, and animal housing systems. New farrowing systems are continuously being developed; however, it is difficult for producers to make decisions without any scientific basis. This study aimed to assess the impact of different farrowing pen layouts on piglet performance, considering parity and season. A total of 546 sows and 9123 piglets were monitored across 36 lactation cycles. Sows were randomly assigned to three farrowing pen layouts (standard, diagonal, and offset) in three rooms (20 sows by room). All farrowing pens had the same space allocations (2.7 m by 1.8 m and 2.1 m by 0.6 m for the sow area). The three types of farrowing pens were blocked by position within the room. Piglet performance traits (percent of stillborns, percent of mortality, percent of overlays, and average daily weight gain: ADG) and sows traits (health and parity) were monitored following US Meat Animal Research Center (USMARC) procedures. Results indicated that treatment, parity, and season influenced some piglet performance traits. The offset farrowing pen had a lower percent of stillborns compared to the standard. No significant differences were observed between the diagonal crate and the other treatments. When evaluating high mortality sow (>two piglets), the offset and standard treatments had a lower percent of overlays. Piglets from first-parity sows had lower ADG than those from higher-parity sows. A higher percent of overlays were observed in Autumn and Summer compared to Spring and Winter, and Summer had lower average daily weight gain than other seasons. The results suggest that modifying the layout (offset), with sows placed further away from the heating source, can reduce the percent of overlays in sows with high mortality (>2 piglets). In addition, the influence of season on the piglet production traits demonstrated the importance of proper management of the environment, even in systems with a certain level of climatic control.

## 1. Introduction

Currently, in the USA swine industry, lactating sows are housed in farrowing crates to protect the piglets from overlays, thus reducing overall pre-weaning mortality (PWM) [1]. However, PWM remains high, averaging over 17.6% [2]. While farrowing crates have been shown to reduce PWM [3,4] these traditional systems are not entirely effective in preventing PWM. Pre-weaning mortality is an economic and animal welfare concern and a complex variable. It is affected by several interconnected factors, such as the sow, the litter, and the environment.

Sow and piglet issues affecting preweaning mortality are interlinked. Genetic selection has led to larger litters [1]; large litters typically have an increased variability in piglet birth weight [5,6]. Higher parity sows have been observed to have better reproductive performance [7]; however, higher parity sows have more overlays and a higher number of stillborns. Both factors have been shown to increase PWM [8]. The smallest piglets in the large litter are at the highest risk, most likely because they are not physiologically mature [9]. In addition, the literature notes that high-parity sows have longer farrowing times [10,11] and less muscle control [12] adding a risk factor for pre-weaning mortality. The most critical time for PWM is the first 72 h after birth, as most mortalities occur during this period [13].

While typical reports from commercial farrowing suggest that the leading causes of piglet mortality are crushing and starvation, these may be secondary causes of the ultimate cause, piglet hypothermia [13]. This suggests the need to increase temperatures for the piglets. However, the management of piglet heating systems is a notable environmental challenge due to the different biological needs of sows and piglets. While sows have a thermal comfort zone between 12 °C and 22 °C [14], preferring temperatures around 14 °C [15], the ideal temperatures for piglet development range around 30 °C to 35 °C [16,17]. Microenvironments within individual crates can be influenced by airflow, seasonal variation, location within the room, supplemental heat source type, position, and age. These variations were studied by Morello et al. (2018) [18]. In an environmentally controlled farrowing room, the spatial distributions of temperature, relative humidity, light intensity, and sound intensity were observed to be up to 9.6 °C, 57%, 3847.3 Lx, 0.87 m s^−1^, and 38.7 dBC among different pens in the same room. Pens positioned near the inlets exhibited the lowest temperatures, suggesting a direct flow of air over these crates. This research underscores that, even in climate-controlled environments, climatic variables within facilities can vary based on seasonal changes and the manipulation of windows and doors. 

These studies show that there are several factors that can influence the performance of piglets before weaning and it is important to continue evaluating different farrowing systems, as this information can help to find better solutions for this phase in order to obtain balanced responses between the welfare and economic viability of production systems. Despite current research on different systems and sizes of farrowing pens, as well as confinement periods for sows [19,20,21], information on different layouts of farrowing pens of the same size and construction materials, and their impact on farrowing parameters, is unclear. To address this gap, it is critical to investigate how this can affect litter performance traits. Changing the position of the sow’s confinement area in the pen can create wider resting area(s) for the piglets and modify the microclimate within the farrowing pen without changing the overall area. The hypothesis is that the new pen arrangements (modifying the space quality) can improve piglet performance.

Therefore, the objectives of this manuscript were to:

(1) Evaluate the temperature distribution within the farrowing pen and how the different pen designs (standard crate—sow crate in the center of the farrowing pen; offset crate—sow crate offset to one side of the farrowing pen; diagonal crate—sow crate positioned diagonally across the farrowing pen) are impacted.
(2) Determine the effect of space quality, season, and sow parity on the PWM and litter performance using the same farrowing pen designs.

## 2. Material and Methods

This study was conducted between January 2020 to May 2021 at the USDA-ARS U.S. Meat Animal Research Center (USMARC) in accordance with federal and institutional regulations regarding proper animal care practices and was approved by the Institutional Animal Care and Use Committee of the US Center for Animal Research (2015–21) (IACUC approval: 1837).

**Figure 1:**
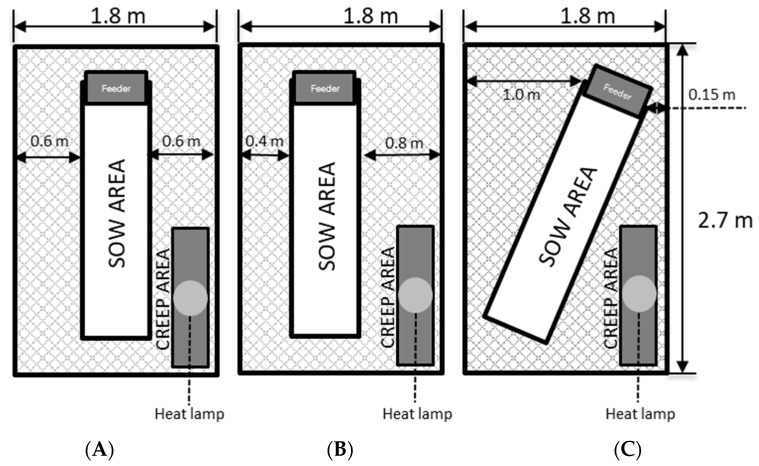
Three experimental farrowing pen layouts used in the experiment: (**A**) standard, (**B**) offset, and (**C**) diagonal. Piglets could circulate throughout the pen (creep area) and the sow movements were limited to only the sow area (2.1 m by 0.6 m).

A total of 546 sows and 9123 piglets were housed in three farrowing pen layouts (Figure 1) that differed in sow crate location (treatments). The animals were randomly distributed and monitored during 36 lactation cycles, and the following piglet production parameters were collected: percent of stillborns, percent of overlays, percent of mortality, and average daily weight gain.

### 2.1. Location and Facilities

The experiment was conducted at the US Department of Agriculture—Agricultural Research Service—US Meat Animal Research Center in Clay Center, Nebraska, USA, located at 40°31′27.39″ N, 98°7′56.69″ W. The collections were carried out in a farrowing facility where the sows were stalled individually in three rooms with a capacity of 20 animals each, totaling 60 farrowing pens.

**Figure 2:**
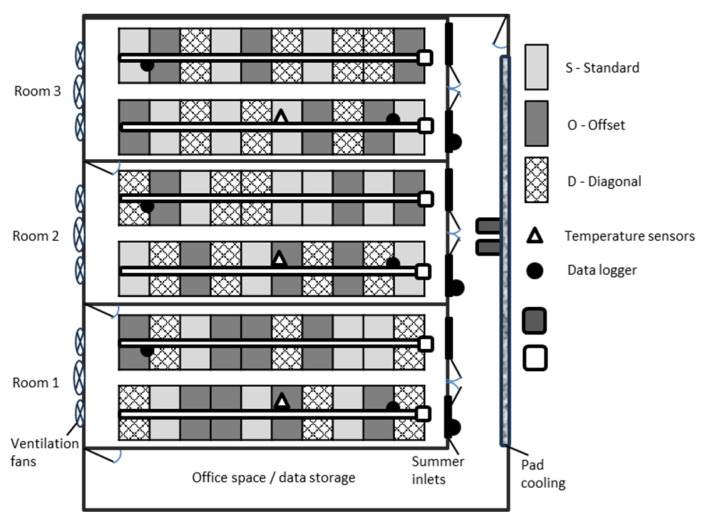
Experimental layout for all cycles. Layout treatments were assigned to a pen so that each position had the same number of treatments tested. Black circle indicates the location of the temperature and relative humidity data loggers. Heat lamps were placed in the back part of the pens for all treatments.

Inside the rooms, the animals were distributed in two rows of ten pens each so that the animals in one row faced the animals in the other row separated by a 1.2 m walkway. The building was climate-controlled with a hallway to precondition the air. Temperature sensors (Figure 2) were used to control the air temperature inside the rooms. The temperature was highest the week of farrowing (set point = 25 °C) and was gradually reduced to 20 °C at the end of the lactation cycle. During the warmest parts of the year, the system incorporated evaporative cooling to help maintain the desired setpoints. Deflectors allowed fresh air into the rooms whenever the exhaust fans located on the opposite end of the room were turned on. During Winter, the hallway shutters were manually closed, and fresh air was then supplied through an air duct suspended from the ceiling the entire length of each room with openings at each sow space. However, in periods of low air temperatures, supplemental forced air heaters (75 KWh) preheat the air in the hallway. Additionally, 10 cm radiant tube heaters (19 KWh) were placed directly above the farrowing pens to provide supplemental heating to the room (Figure 2). 

The installation consisted of individual feeding troughs (Sow Max AdLib Sow Feeder—Hog Slat) that were arranged in the direction of the central corridor of each room. 

The flooring was 1 cm wide—T-slat through the space. Waste management was handled using a shallow pit and a manual flush system using a 1900 L manual water dump for each set of 10 crates that operated twice a day.

### 2.2. Farrowing Pen Layouts

The farrowing pen had the same size regardless of the treatments with 1.8 m (W) × 2.7 m (L) × 1.2 m (H), and the farrowing crate (sow confinement space) was 0.6 m (L) × 2.1 m (W) × 1.5 m (H). The entire floor area was metal slats, with a slat width of 8.5 mm and a gap of 9.5 mm between the slats (TriDEK, Hog Slat, Inc.; Newton Grove, NC, USA). The slats had non-slip grip cutouts and were the same throughout the entire pen. A rubber mat was placed under the heat lamp area. The treatments involved three types of farrowing pens (standard, diagonal, and offset) differentiated by the positioning of the sow crate in the area. For the standard layout, the sow crate was placed in the middle of the pen; in the diagonal layout, the sow crate was placed diagonally in the pen, and in the offset layout the crate was centered 0.2 m from the center of the pen. The farrowing pen layouts are shown in Figure 1.

Supplemental heating for the piglets had a 175 W ceramic infrared lamp placed over a rubber mat (1.1 × 0.3 m) positioned at the rear of each pen. Each lamp was covered by metal lamp protectors and suspended so that the average temperature on the mat was around 36.0 °C. The heat lamps started operating approximately two days before the expected farrowing date and remained on until the piglets were approximately 21 days old. All lamps operated connected to a thermostat which automatically turned off if the ambient air temperature exceeded 5.5 °C above the nominal ambient temperature (approximately 25.0 °C).

The treatments were present in the same number and were randomly distributed within the rooms. Positions were maintained throughout the experiment and all available pens were used for data collection (Figure 2).

### 2.3. Animals and Management

The experiment had 36 data collection cycles. In each cycle, a group of up to 20 pregnant sows (Landrace × Yorkshire) of different parities (1, 2, 3, and 4) was monitored for 32 days. After this period, the group left the room, which was cleaned and disinfected for another group of 20 sows to enter the study. Each group of sows in the room represented a cycle. The mean weight of the sows was 191.8 ± 29.8 kg, and the mean number of piglets born was approximately 14.0 ± 3.9 piglets/sow. Some sows entered the experiment more than once.

The sows were fed with a corn-soy ration (3222 kcal/kg of metabolizable energy, 19.5% protein, 3.93% fat, 7.04% neutral detergent fiber, and 1.18% total lysine) once a day for up to three days after farrowing and then ad libitum feed was provided. Feed was also provided to piglets from approximately 21 days of age until weaning at 28 days. Drinking water was provided as ad libitum to piglets and sows using two nipple drinkers placed at 13 cm and 86 cm off the ground.

Trained animal caretakers followed standard operating procedures: drying mineral powder was distributed onto the mats under supplemental heat lamps prior to piglet birth; piglets were weighed and ear-tagged on day one (between 18–30 h after birth); tails docked, needle teeth clipped, castration, and iron injections were administered to the piglets three days after birth. As necessary for achieving consistent litter sizes, piglets were cross-fostered. More details of materials and handling practices commonly used in the facilities where the experiment was conducted can be found in [20].

### 2.4. Data Collection 

#### 2.4.1. Characterization of the Climate Environment

To monitor the microclimate of the installation, two data loggers (XR440, Pace Scientific, Boone, NC, USA) were installed in each of the rooms suspended 1.2 m from the floor. One data logger was installed over the first pens at the hallway end of the room and the other at the opposite end of the room where ventilation fans resided. These devices recorded relative humidity and dry-bulb temperature (DBT) every 5 min throughout the experiment. From these data, the pocket logger software (Verison 3.3; Pace Scientific, Boone, NC, USA) was used to calculate and output dew point temperature.

#### 2.4.2. Production Data 

Trained caretakers recorded all important information related to the production and health of the sows and piglets. Caretakers noted the following sow health issues during the study: infected uterine, mastitis, dystocia routine pull, open wound, undiagnosed illness, abscessed, lame, infected joint, and seroma. Litter performance traits were collected on each litter and included: the number born, number live at birth, number of mummies, number of stillborns, number of overlays, other causes of death (euthanasia), total mortality, and total weaned by sow. Piglets were weighed individually within 24 h after birth and one day before weaning. In addition, the lactation period was accounted for. With this information, the percent of stillborns (PS), percent of overlays (PO), pre-weaning mortality (PWM), and average daily weight gain (ADG), were calculated according to the Equations (1), (2), (3) and (4), respectively.

(1) $$\mathrm{PS}=\frac{\mathrm{Number}\;\mathrm{of}\;\mathrm{stillborns}}{\mathrm{Total}\;\mathrm{number}\;\mathrm{of}\;\mathrm{piglets}\;\mathrm{born}}*100$$

(2) $$\mathrm{PO}=\frac{\mathrm{Number}\;\mathrm{of}\;\mathrm{overlays}}{\mathrm{Total}\;\mathrm{number}\;\mathrm{of}\;\mathrm{piglets}\;\mathrm{born}\pm \mathrm{fostered}\;}*100$$

(3) $$\mathrm{PWM}=\frac{\mathrm{Number}\;\mathrm{dead}}{\mathrm{Total}\;\mathrm{number}\;\mathrm{born}\pm \mathrm{fostered}}*100$$

(4) $$\mathrm{ADG}=\frac{\mathrm{Weight}\;\mathrm{at}\;\mathrm{weaning}-\mathrm{Weight}\;\mathrm{at}\;\mathrm{birth}}{\frac{\mathrm{Days}\;\mathrm{of}\;\mathrm{lactation}}{\mathrm{Number}\;\mathrm{of}\;\mathrm{piglets}\;\mathrm{at}\;\mathrm{weaning}}}$$

#### 2.4.3. Statistical Analysis 

Preliminary data were collected to determine the number of samples required. Based on the analysis, to achieve 85% statistical power in detecting differences in the percent of overlays between treatments, it was considered that a minimum of 81 sows per treatment was needed to determine differences. All data were analyzed using the MIXED procedure of SAS (SAS Institute Inc., Cary, NC, USA). Initially, all data were analyzed for the presence of disparate information (‘outliers’) and normality of residuals (Shapiro–Wilk) using the Univariate procedure of SAS. Individual observations were considered as outliers when they were more than three standard deviations away from the mean. When the assumption of normality for residuals was violated, data were transformed (log transformation) to conform to a normal distribution. Treatment was the fixed effect investigated in the model. Percent of stillborns (PS) (Equation (5)), percent of overlays (PO) (Equation (6)), and average daily weight gain (ADG) (Equation (7)) were the piglet performance traits investigated according to the fixed effects: farrowing pen layout, parity, season, and all their interactions. The litter was the experimental unit considered for PS and PO, and the piglet was the experimental unit for ADG. Sow health was used as a random effect and recorded as a two-level categorical variable (had/did not have health problems during the experiment). The total number of pigs born was included as a covariable to the PS model. The total number of pigs raised by the sow (litter size) was covariable to the PO and ADG models. Since PS and PO were not normally distributed, a logarithm function was used to transform the data prior to analysis. Comparisons of means were performed by ANOVA and student t-test, using the Mixed procedure at a significance level of *p* < 0.10.

An additional analysis was performed to evaluate the impact of farrowing pen layout on high-mortality litters. This analysis used only sows with PWM greater than two piglets from the data. These data represent about 42% of the total data, and the distribution across pen layouts is similar. PS, PO, and ADG were compared according to treatments.

Log(PS) = β_0_ + β_1_(Farrowing Pen Layout) + β_2_(Parity) + β_3_(Season) + β_4_(Interaction) + β_5_(Total Number Born) + Random Effect (Sow Health) + ϵ (5)

Log(PO) = β_0_ + β_1_(Farrowing Pen Layout) + β_2_(Parity) + β_3_(Season) + β_4_(Interaction) + β_5_(Litter Size) + Random Effect (Sow Health) + ϵ (6)

ADG = β_0_ + β_1_(Farrowing Pen Layout) + β_2_(Parity) + β_3_(Season) + β_4_(Interaction) + β_5_(Litter Size) + Random Effect (Sow Health) + ϵ (7)

where:

β_0_, β_1_, β_2_, β_3_, β_4_, and β_5_ are the coefficients associated with the intercept, fixed effects, and covariables.

ϵ is the error term.

## 3. Results

### 3.1. Description of the Thermal Environment

**Figure 3:**
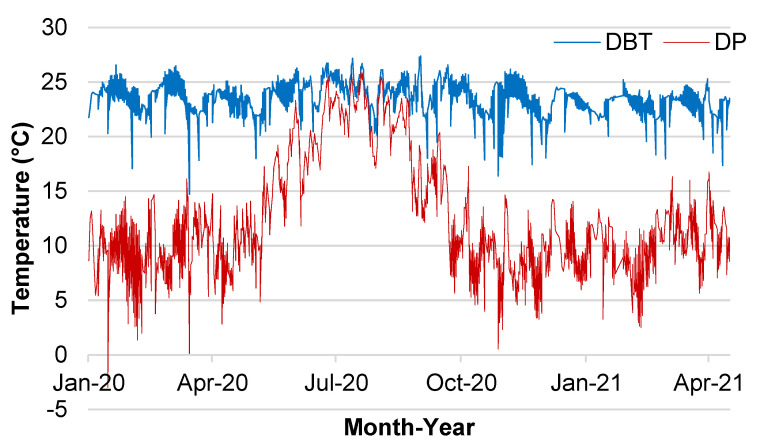
Dry-bulb temperature (DBT) and dew-point (DP) distribution from the data were collected on 36 cycles of sows entering the farrowing facility. Three farrowing rooms were used, and collection occurred from January 2020 to May 2021.

During the collection period, the values of the environmental variables were close in the three rooms where the average dry-bulb temperature and the mean relative humidity were below 25 °C and 80%. Despite the dry-bulb temperature having little variation, the dew-point temperature varied considerably (Table 1, Figure 3). The mean DP values recorded in each season were 23.3 ± 0.7 (Autumn), 23.3 ± 0.7 (Spring), 24. 3 ± 1.2 (Summer), 23.4 ± 0.4 (Winter).

The mean values of DBT, RH, and DP showed statistical differences between seasons (Table 2). DBT was different for all seasons (*p* < 0.01), and the mean DBT value was highest in summer (24.6 ± 0.1 °C). There was no difference between the mean RH value in Autumn (42.5 ± 0.6%) and Winter (43.6 ± 0.5%), but their values were different (*p* < 0.01) than those recorded for spring (55.1 ± 0.6%) and summer (82.4 ± 0.7%). The same happened for the mean DP values, which were equal between Autumn (9.5 ± 0.2 °C) and Winter (9.6 ± 0.1 °C) and different (*p* < 0.01) from the means recorded for spring (13.5 ± 0.2 °C) and summer (21.2 ± 0.2 °C).

### 3.2. Thermal Distribution in the Farrowing Pen Layouts

**Figure 4:**
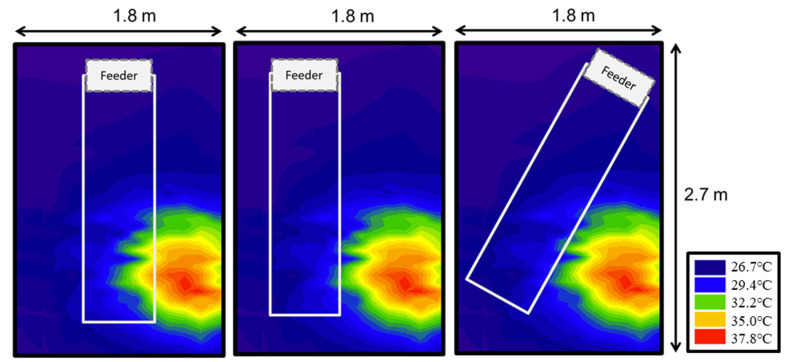
Thermal distribution in the farrowing pen layouts obtained with a thermal camera.

To evaluate the thermal distribution in the farrowing pens, an image was obtained with a thermal camera (FLIR–T860, Teledyne FLIR, Solutions 110 Lowell Rd. Hudson, NH, USA) and superimposed on the three evaluated layouts (standard, offset, and diagonal) (Figure 4). In the images, the red, yellow, and green colors represent the highest temperature values (32.2 to 37.8 °C) and represent the heat emitted by the supplementary heating lamp. In the standard treatment, the heat emitted by the lamp invades the sow area, while in the offset and diagonal treatments, this supplementary heat does not impact the sow area.

### 3.3. Treatments and Productive Parameters

A total of 74 litters were eliminated from the data set due to causes not associated with the experimental treatments. These litters were associated with sows with injuries or euthanized, and changes in sows between treatments were removed from the experiment. Additionally, sows with 100% stillborns and treatments with more than 80% euthanized piglets were excluded from the dataset. Parity was similarly distributed between treatments. Of the 651 replicates in the study, 35.5% of the sows were from the first parity, 30.3% from the second, 19.5% from the third, and 14.6% from the fourth. A total of 9123 piglets were born and the average by treatment was 14.2 ± 3.9 piglets/sow (standard), 14.0 ± 3.7 piglets/sow (diagonal), and 13.7 ± 4.1 piglets/sow (offset). A summary of all production data by treatment can be seen in Table 3. The PWM was over 16.0% for all treatments and the PO for the standard, diagonal, and offset treatments was 6.0%, 5.5%, and 6.2%, respectively. Standard treatment had a greater coefficient of variation than the diagonal and offset treatments, both for PWM (81.5%) and for the PO (162.7%). The ADG of piglets was close for all treatments (mean = 238.2 ± 32.4 g) but the coefficient of variation for the diagonal treatment was lower (12.8%) than for offset (13. 1%) and standard (14.1%).

No interaction effects were observed between the variables of farrowing pen treatments, parity, and season on productive traits (PS, PO, and ADG). The effects of farrowing pen layout, season, and parity on productive traits can be seen in Table 4. Farrowing pen treatments only presented statistical differences for PS (*p* = 0.06) with higher values for standard (5.8 ± 0.9%) in comparison with the offset treatment (5.3 ± 0.8%). Season influenced PO (*p* = 0.01), and ADG (*p* < 0.01). PO was higher in the Autumn (8.5 ± 0.8%) and Summer (8.2 ± 0.9%) than in the Spring (5.6 ± 0.7%) and Winter (4.6 ± 0.7%). ADG was lower in the Summer (231.6 ± 3.03 g) compared to the other seasons (mean = 243.7 ± 2.3 g). Parity affected ADG (*p* < 0.01). Piglets from first parity sows had lower ADG (218.6 ± 2.06 g) than the second (243.9 ± 2.2 g), third (251.7 ± 2.6 g), and fourth (248.5 ± 3.1 g) parities. 

When data from sows with mortality greater than two piglets were observed (Table 5 and Table 6), treatment influenced PO (*p* = 0.09) (Table 6). In this case, the standard treatment presented higher PO (11.3 ± 1.1%) than the offset treatment (10.7 ± 1.1%). 

## 4. Discussion

### 4.1. Treatment and Litter Performance Traits

Piglet performance traits are intricately linked to the animals’ performance and physiology throughout the lactation period. Therefore, parameters such as PS, PO, and ADG are commonly assessed to characterize the efficiency of the production system. In the present study, the overall data revealed an average PWM of 17.8%. This PWM value falls below the 25% figure reported by Marchant et al. (2001) [3], in a study on PWM attributed to crushing in sows housed in free pens during the initial week of lactation. Conversely, it exceeds the mean PWM reported by Heidinger et al. (2022) [19], at 13.7%, who examined various periods of sow confinement in farrowing pens.

The expectation was that adjustments to the farrowing pen layout, relocating sows away from the heat source, and providing an alternative free resting area for piglets could enhance their performance. Thermal distribution within the farrowing pen (Figure 4) illustrates that, in the standard treatment, the sow area experiences the most impact from the heat lamp, elevating the temperature to levels exceeding 32.0 °C, beyond the optimal thermal comfort range for sows (12.0 to 22.0 °C) [23]. In this study, when analyzing overall data, no significant differences were observed in PO, and ADG between the treatments. The only piglet performance trait exhibiting a statistical difference was PS, where the offset treatment displayed a lower PS compared to the standard treatment. This difference may be attributed to the altered layout promoting a more comfortable microclimate for the sows, potentially influencing the farrowing process. 

Wegner et al. (2016) [24], conducted a study on the influence of temperature and the Temperature and Humidity Index on the reproductive performance of sows during the Summer months. Their findings revealed that as climatic variables increased, there was a corresponding decrease in the number of piglets born alive and an increase in the number of stillborns. Notably, these observations were made in an experiment conducted in a moderate climate, with the authors recording an average temperature consistently below or equal to 16 °C throughout all three evaluated summers. In the current study, the average temperature across all seasons ranged from 23.2 °C (Winter) to 24.6 °C (Summer) (Table 2). However, it is crucial to highlight that the physiological or behavioral parameters of the sows, which could validate this observed trend, were not investigated in this study. Future research focusing on these aspects among various proposed alternative pens may provide insights into the identified effects. Despite the difference in layouts, examined in this study maintained an equal yet expanded size compared to a traditional farrowing pen. Additionally, exploring these same treatments in a traditional, smaller-sized farrowing pen would be valuable to assess whether relocating the sow from the heating source has an impact on production traits.

In the evaluation of sows with high mortality (2 piglets), the results showed that the offset treatment had lower PO than the standard. This observation may be attributed to the fact that the selected sows in this category had notably larger litters, averaging 16.2 piglets per sow, in contrast to the general data with an average of 14.0 piglets per sow. The presence of a larger litter tends to intensify competition for nursing space and occupies more resting areas, thereby posing an increased risk of crushing for the animals involved. This indicates that as litters become larger, alternative farrowing pen designs such as the offset design become increasingly more important.

Leonard et al. (2021) [20], investigated the impact of the farrowing pen size and the number of heat lamps on piglet performance. Their investigation compared traditional farrowing pens, pens with expanded piglet rest areas, and pens with expanded areas for sows and piglets. The authors reported no differences between treatments in percent of mortality, percent of overlays, number of piglets born alive, or the total number of piglets weaned per sow. Similar to the current study, the authors identified statistical differences in the percent of stillborns among different pen layouts. The authors highlighted the importance not only of the amount of space but also of its usability. Nevertheless, in the present study, when considering this space quality, no significant differences were found in the litter performance traits across treatments using general data. Conversely, when focusing on high-mortality sows (>2 piglets), the offset treatment had a lower PO compared to the standard pen layout. This suggests that, particularly for sows with larger litters, a simple modification of the farrowing pen layout and the provision of a wider piglet resting area on one side of the sow could contribute to mitigating mortalities caused by overlays.

Moustsen et al. (2013) [21], evaluated four distinct periods of sow confinement: sows released throughout the entire lactation period, sows confined in crates from day 0 to 4 postpartum, sows confined from day 0 to 7 postpartum, and sows crated from introduction to the pen until day 7. The findings indicated a lower piglet mortality rate when sows were confined, as opposed to sows without any movement restraint. However, there were no significant differences in piglet mortality observed among the other confinement periods that were evaluated.

In addition to the aforementioned investigations, other studies have reported a reduction in piglet mortality with the confinement of sows [4,25]. Despite the increasing consideration for adopting systems without the confinement of sows, it enables the assessment of diverse physiological and environmental aspects in productive parameters. Individual systems of this nature incorporate standardized protocols conducive to individual animal monitoring. While a consensus on the optimal type or management strategy has yet to emerge, research involving sows in crates is pivotal for acquiring insights to enhance system management. Positive effects on piglet performance have been observed when sows are housed in farrowing crates compared to free farrowing. Furthermore, studies highlight that most mortalities occur within the initial week after farrowing. Combining this information with the current study’s findings suggests that the adoption of larger farrowing pens, with the crate further away from the heating source and, potentially, for a shorter duration, could present a more sustainable alternative compared to conventional farrowing pen systems. As previously mentioned, additional studies exploring the interplay between animal behavior and productive parameters can contribute to a more comprehensive investigation of the proposed systems. Moreover, investigations considering environmental perspectives and seasonal variations can offer pertinent insights into both systems and animal welfare.

### 4.2. Season and Parity Affecting Litter Performance Traits

Sow parity and the season of the year were also investigated as factors influencing piglet performance traits, as existing studies suggest a direct correlation between high parity and elevated temperatures with diminished piglet performance [10,11,24,26]. In the present study, parity did not exhibit a significant impact on PS or PO, but it did influence ADG, with first-parity sows having piglets with lower ADG compared to sows with higher parities (Table 4). Leonard et al. (2021) [20], explored piglet performance across different farrowing pen treatments and noted that parity not only affected ADG but also influenced the percent of mortality and the percent of overlays. The authors reported that fourth-parity sows had a higher percent of overlays than first-parity and a higher percent of mortality than first- and second-parities. Additionally, concerning ADG, piglets from first-parity sows displayed lower ADG than those from higher-parity sows. 

Li et al. (2023) [27], studied the effect of maternal behaviors (postural, nursing, defense, and sow-piglet communication) of primiparous and multiparous sows on litter weight and number of piglets crushed. The study revealed that multiparous sows exhibited good maternal behavior in terms of time spent in a specific posture, feeding arrangement, care for lying down, and response to the “piglets’ request for help”. No discernible effects were noted on litter weight and the number of crushed piglets, as these traits displayed no significant variations between primiparous and multiparous sows. Although the aforementioned study did not show any differences in production parameters, the better maternal ability of multiparous sows may explain the difference observed in ADG between first-parity and higher-parity sows in this study. Additionally, considering their youth and ongoing growth and development, primiparous sows may allocate less time for nursing or utilize part of the energy that could be directed toward milk production for their own development.

In this study, even within a controlled environment, the influence of the season on the PO and ADG was observed (Table 4). Autumn and Summer presented higher PO values than Spring and Winter, while Summer demonstrated lower ADG compared to the other seasons. During the Summer, one explanation for these differences could be the elevated RH resulting from the activation of evaporative cooling pads to mitigate the high air temperature (Table 2). Increased RH limits the sow’s capacity to dissipate heat through evaporation in environments with elevated temperatures [28]. Consequently, an increase in RH may induce thermal discomfort in sows and alter their behavior, thereby potentially influencing piglet performance. Milk production and feed intake exhibit a close relationship and are susceptible to the effects of hyperthermia given their direct connection to the nutritional well-being of sows [13]. In thermal discomfort, sows tend to reduce food and water intake. A meta-analysis of literature data conducted by Dourmad et al. (2022) [29] revealed that lactating sows experienced a 36% reduction in feed intake and a 20% decrease in milk production when temperatures ranged between 22 °C and 32 °C. Additionally, hyperthermia can impact the frequency and duration of postural changes in sows [11], a factor directly linked to their availability for nursing and the PO.

An additional justification for elevated PO values observed during Autumn lies in the manual management of the air inlets, a factor directly influencing the heat flow in the facility. In the Midwest of the USA, in the Autumn, the temperature fluctuations require continuous adjustments to the inlets. For example, during the study, on 7 September, the highest temperature was 35.5 °C (ranging from 16.2 °C to 22 °C indoors) and in two days (9 September), the highest temperature was only 7.8 °C (ranging from 11.5 °C to 20.3 °C indoors). Thus, making it difficult to manually adjust the ventilation from summertime tunnel ventilation to wintertime ceiling-type ventilation. Therefore, resulting in improper air inlet regulation, and plausible sow and piglets’ thermal stress. In colder periods, the piglets tried to warm themselves closer to the sows. The options available to piglets include artificial heating sources or seeking warmth in the vicinity of the sow [30]. In addition, it is part of their natural behavior to stay close to the sow in the first few days after birth [31,32]. Inadequate management of these systems can adversely affect piglet performance, prompting them to seek warmth near the sow and increasing the risk of crushing. On the other hand, in warmer periods, supplemental heat lamps are switched off and, in this situation, there is no difference between lying next to the sow and under the heating lamp. Therefore, the piglets’ behavior may have changed, causing them to lie down closer to the sow, increasing the risk of overlays.

Studying the behavior of sows, piglet location, and assessing stress are important and complementary information that should be investigated in future studies. 

## 5. Conclusions

The aim of this study was to evaluate the effect of different farrowing pen layouts, parity, and season on piglet performance traits before weaning. Space quality was evaluated by comparing three farrowing pen layouts. The standard pen with the sow in the center—creating long narrow piglet areas under the supplemental heat lamp. Two alternative pen designs—diagonal (sow crate placed diagonally within the pen) and offset (sow crate placed on one side of the pen) both created for a wider piglet area under the heat lamps. In the evaluation of the thermal distribution, using the thermal camera, it was determined that the alternative designs created a better temperature separation between the sow and the piglet areas.

While pen design only impacted the percent of stillborns, with the offset crates having lower stillborns than the standard crate and the diagonal crates being in between. The pen design impacted percent overlays when evaluating only high-mortality sows (losses > 2 piglets). It was noted that high-mortality sows averaged 16.2 piglets per sow compared to an average of 14.0 piglets per sow in the general population. Therefore, it was concluded that pen design may become more important in populations of sows with larger litters or in the future if litter sizes continue to increase. Although the diagonal crate showed good temperature separation between the sow and piglet areas, there was no advantage in using this design to improve piglet performance.

First parity sows had piglets with lower ADG than multiparous sows; however, parity did not influence the percent of stillborns or overlays. A higher percent of overlays was observed in Autumn and Summer compared to Spring and Winter; it was hypothesized this could be due to the higher temperatures in the Summer and the management of the inlet in Autumn. In addition, a lower ADG was observed during the Summer than in the other seasons, again possibly due to the higher maximum temperatures. 

The results of this study demonstrate that adopting a different farrowing pen geometry can improve piglet performance by reducing the number of stillborns and the number of overlays in high-mortality sows. In addition, this study demonstrates the importance of efficient climatic management, even in systems with a certain degree of automation since differences were found in production parameters between seasons. Further studies focusing on the behavior of the sows and piglets in these three pen designs will contribute to the understanding of the effects of season and litter size.

## Figures and Tables

**Table 1:** Mean and standard deviation (stdev) of the environmental variables and Temperature Humidity Index obtained during the experiment. Parameters obtained from the average daily values monitored during the experiment.

Parameter	Mean ± Stdev	Min	Max
Dry-Bulb Temperature (°C)	23.6 ± 1.3	14.7	30.4
Wet Bulb Temperature (°C)	16.9 ± 3.0	13.9	25.2
Relative Humidity (%)	52.8 ± 5.0	26.3	97.1
Dew Point (°C)	12.5 ± 1.5	3.3	26.8
Enthalpy (kJ/kg)	51.1 ± 10.5	41.3	82.1
Temperature Humidity Index *	69.7 ± 2.7	67.1	78.0

* Temperature and Humidity Index (THI) calculated with the equation THI = 0.72 × (DBT + WBT) + 40.6 [22] where the DBT is the dry-bulb temperature and WBT is the web bult temperature.

**Table 2:** Values (mean ± standard error (SE)) of dry-bulb temperature (DBT), relative humidity (RH), and dew point (DP) by season.

Season	Mean ± SE		
	DBT (°C)	RH (%)	DP (°C)
Spring	23.6 ± 0.1 ^a^	55.1 ± 0.6 ^a^	13.5 ± 0.2 ^a^
Summer	24.6 ± 0.1 ^b^	82.4 ± 0.7 ^b^	21.2 ± 0.2 ^b^
Autumn	23.4 ± 0.1 ^c^	42.5 ± 0.6 ^c^	9.5 ± 0.2 ^c^
Winter	23.2 ± 0.5 ^d^	43.6 ± 0.5 ^c^	9.6 ± 0.1 ^c^

^a,b,c,d^ Columns were differing superscripts were significantly different (*p* < 0.05).

**Table 3:** Summary of production data by treatment: mean, standard deviation (SD), and coefficient of variation (CV). Values obtained in relation to the general data collected in the experiment.

Parameter	Standard (*n* = 210)			Diagonal (*n* = 216)			Offset (*n* = 225)		
	Mean	SD	CV	Mean	SD	CV	Mean	SD	CV
Parity	2.2	1.0	47.6	2.1	1.1	52.0	2.1	1.1	49.4
Lactation length (days)	26.3	2.1	7.9	26.4	1.8	6.8	26.4	2.2	8.2
Number born	14.2	3.9	27.4	14.0	3.7	26.8	13.7	4.1	30.0
Number live at birth	12.9	3.6	27.9	13.0	3.4	26.5	12.7	3.7	28.9
Litter size	11.6	1.8	15.9	11.7	1.6	13.6	11. 9	1.7	14.7
Total weaned by sow	10.9	1.8	16.4	11.1	1.5	13.9	11.1	1.4	12.8
Pre weaning mortality ^a^	18.8	15.3	81.5	17.8	14.0	79.0	17.0	12.6	74.0
Percent mummies ^b^	2.9	6.8	231.2	1.5	3.7	255.2	1.9	5.0	265.8
Percent stillborns ^c^	5.3	8.9	166.6	5.5	7.9	144.2	4.9	6.8	139.9
Percent overlays ^d^	6.0	9.8	162.7	5.5	7.8	143.0	6.2	8.0	130.4
Percent other causes ^e^	5.7	8.7	152.7	6.3	8.9	140.6	4.9	6.9	141.3
ADG ^f^	237.8	33.5	14.1	239.6	30.7	12.8	237.3	31.1	13.1

^a^ Calculated using the number of mortalities (number of mummies, number of stillborns, number of overlays, and other causes of death) divided by number of piglets live a birth ± cross-fostered piglets; ^b^ Calculated using the number of mummies divided by number of piglets born; ^c^ Calculated using the number of stillborns divided by number of piglets born; ^d^ Calculated using the number of overlays divided by number of piglets live a birth ± cross-fostered piglets; ^e^ Calculated using the number of mortalities by other causes (diarrhea, euthanasia) divided by number of piglets live at birth ± cross-fostered piglets; ^f^ Calculated subtracting the piglet’s weight (g) at birth from weaning divided by the number of piglets at weaning, and by the days of lactation.

**Table 4:** LS mean values of the piglet performance traits (percent stillborns (PS), percent overlays (PO), and average daily gain (ADG)) by farrowing pen layout, season, and parity.

**Parameter**	**Treatment**				**Effects (*p*)**
	**Standard**	**Diagonal**		**Offset**	
PS	5.8 ± 0.9 ^a^	5.6 ± 0.9 ^a,b^		5.35 ± 0.8 ^b^	0.06
PO	7.3 ± 0.7	6.2 ± 0.7		6.7 ± 0.7	0.28
ADG	237.9 ± 2.2	242.2 ± 2.2		242.0 ± 2.1	0.29
**Parameter**	**Season**				**Effects (*p*)**
	**Spring**	**Summer**	**Autumn**	**Winter**	
PS	5.3 ± 0.9	6.0 ± 1.0	5.0 ± 0.9	6.0 ± 0.8	0.11
PO	5.6 ± 0.7 ^a^	8.2 ± 0.9 ^b^	8.5 ± 0.8 ^b^	4.6 ± 0.67 ^a^	0.01
ADG	243.3 ± 2.3 ^a^	231.6 ± 3.0 ^b^	242.0 ± 2.5 ^a^	245.8 ± 2.1 ^a^	<0.01
**Parameter**	**Parity**				**Effects (*p*)**
	**1**	**2**	**3**	**4**	
PS	5.7 ± 0.8	5.3 ± 0.8	4.4 ± 1.0	6.9 ± 1.1	0.11
PO	5.3 ± 0.7	6.8 ± 0.7	7.3 ± 0.8	7.5 ± 0.9	0.28
ADG	218.6 ± 2.1 ^a^	243.9 ± 2.2 ^b^	251.7 ± 2.6 ^c^	248.5 ± 3.1 ^b,c^	<0.01

(PS) Calculated using the number of stillborns divided by number of piglets born; (PO) Calculated using the number of overlays divided by number of piglets live a birth ± cross-fostered piglets; (ADG) Calculated subtracting the piglets’ weight (g) at birth from weaning divided by the number of piglets at weaning, and the days of lactation. ^a,b,c^ Different letters between columns indicate statistical differences between mean values (*p* < 0.10).

**Table 5:** Summary of production data by treatment: mean, standard deviation (SD), and coefficient of variation (CV). Values obtained in relation to the selected data (excluding the sows with mortality less or equal to 2 piglets).

Parameter	Standard (*n* = 97)			Diagonal (*n* = 92)			Offset (*n* = 87)		
	Mean	SD	CV	Mean	SD	CV	Mean	SD	CV
Parity	2.3	1.1	48.0	2.3	1.1	50.3	2.4	1.1	43.8
Lactation length	26.4	2.0	7.4	26.2	1.9	7.3	26.7	2.1	8.0
Number born	16.1	3.2	20.1	16.0	3.0	18.9	16.5	3.1	19.0
Number live at birth	13.9	3.4	24.3	14.1	2.3	21.1	14.6	3.1	21.2
Litter size	11.6	2.0	17.6	11.7	1.8	15.5	12.4	1.9	15.4
Total weaned by sow	10.5	1. 9	18.0	10.6	1.6	14.7	11.0	1.3	12.2
Pre weaning mortality ^a^	31.4	12.7	40.5	31.3	9.4	30.1	29.9	8.1	27.2
Percent mummies ^b^	4.7	8.6	182.0	2.2	4.1	184.8	3.9	7.2	183.8
Percent stillborns ^c^	9.0	10.8	119.2	9.9	9.2	92.5	7.9	7.4	94.1
Percent overlays ^d^	10.0	12.4	123.4	9.3	6.4	101.3	11.3	9.4	82.9
Percent other causes ^e^	9.9	10.6	107.5	11.7	10.0	89.8	8.5	8.4	99.5
ADG ^f^	236.4	32.3	13.7	235.5	29.7	12.6	237.2	27.9	11.7

^a^ Calculated using the number of mortalities (number of mummies, number of stillborns, number of overlays, and other causes of death) divided by number of piglets live a birth ± cross-fostered piglets; ^b^ Calculated using the number of mummies divided by number of piglets born; ^c^ Calculated using the number of stillborns divided by number of piglets born; ^d^ Calculated using the number of overlays divided by number of piglets live a birth ± cross-fostered piglets; ^e^ Calculated using the number of mortalities by other causes (diarrhea, euthanasia) divided by number of piglets live at birth ± cross-fostered piglets; ^f^ Calculated subtracting the piglet’s weight (g) at birth from weaning divided by the number of piglets at weaning, and by the days of lactation.

**Table 6:** LS mean value of the piglet performance traits (percent stillborns (PS), percent overlays (PO), and average daily gain (ADG)) by farrowing pen treatment, season, and parity. Values obtained in relation to the selected data (excluding the sows with mortality less or equal to 2 piglets).

**Parameter**	**Treatment**				**Effects (*p*)**
	**Standard**	**Diagonal**		**Offset**	
PS	9.3 ± 1.0	9.9 ± 1.2		7.8 ± 1.1	0.13
PO	11.3 ± 1.1 ^a^	10.7 ± 1.2 ^a,b^		10.7 ± 1.1 ^b^	0.09
ADG	235.0 ± 3.9	235.9 ± 4.4		239.5 ± 4.2	0.58
**Parameter**	**Season**				**Effects (*p*)**
	**Spring**	**Summer**	**Autumn**	**Winter**	
PS	8. 6 ± 1.1	9.1 ± 1.4	8.4 ± 1. 4	9.9 ± 1.2	0.44
PO	8.6 ± 1.1 ^a^	14.2 ± 1.5 ^b^	12.7 ± 1.4 ^b^	8.1 ± 1.2 ^a,b^	<0.01
ADG	237.1 ± 4.1 ^a^	228.1 ± 5.0 ^b^	237.7 ± 5.0 ^a,b,c^	244.2 ± 4.2 ^a,c^	0.04
**Parameter**	**Parity**				**Effects (*p*)**
	**1**	**2**	**3**	**4**	
PS	9.7 ± 1.3 ^a,c^	8.0 ± 1.1 ^b^	8.2 ± 1.5 ^a,b^	10.2 ± 1.6 ^c^	0.03
PO	10.4 ± 1.3	11.0 ± 1.1	12.2 ± 1.5	9.9 ± 1.4	0.94
ADG	215.0 ± 4.2 ^a^	241.3 ± 4.1 ^b^	251.7 ± 2.6 ^c^	248.5 ± 3.1 ^b,c^	<0.01

(PS) Calculated using the number of stillborns divided by number of piglets born; (PO) Calculated using the number of overlays divided by number of piglets live a birth ± cross-fostered piglets; (ADG) Calculated subtracting the piglets’ weight (g) at birth from weaning divided by the number of piglets at weaning, and the days of lactation. ^a,b,c^ Different letters between columns indicate statistical differences between mean values (*p* < 0.10).

## Data Availability

The data presented in this study are available on request from the corresponding author. The data are not publicly available because the author want to ensure proper interpretation of the data.
